# Internet Delivered Support for Tobacco Control in Dental Practice: Randomized Controlled Trial

**DOI:** 10.2196/jmir.1095

**Published:** 2008-11-04

**Authors:** Thomas K Houston, Joshua S Richman, Midge N Ray, Jeroan J Allison, Gregg H Gilbert, Richard M Shewchuk, Connie L Kohler, Catarina I Kiefe, DPBRN Collaborative Group

**Affiliations:** ^8^Names are listed in dental investigator group at Dental PBRN.orgBirminghamALUSA; ^7^Department of Health BehaviorSchool of Public HealthUniversity of Alabama at BirminghamBirminghamALUSA; ^6^Department of Diagnostic SciencesSchool of DentistryUniversity of Alabama at BirminghamBirminghamALUSA; ^5^Department of Health Services AdministrationUniversity of Alabama at BirminghamBirminghamALUSA; ^4^Division of Preventive MedicineUniversity of Alabama at BirminghamBirminghamALUSA; ^3^Center for Outcomes and Effectiveness ResearchUniversity of Alabama at BirminghamBirminghamALUSA; ^2^Division of General Internal MedicineUniversity of Alabama at BirminghamBirminghamALUSA; ^1^Surgical and Medical Acute care and Advanced illness Research and Transition sciences (SMAART) Centera VA HSR&D REAPBirmingham VA Medical CenterBirminghamALUSA

**Keywords:** Smoking cessation, Internet, general practice, dental, randomized controlled trial, health services research

## Abstract

**Background:**

The dental visit is a unique opportunity for tobacco control. Despite evidence of effectiveness in dental settings, brief provider-delivered cessation advice is underutilized.

**Objective:**

To evaluate an Internet-delivered intervention designed to increase implementation of brief provider advice for tobacco cessation in dental practice settings.

**Methods:**

Dental practices (N = 190) were randomized to the intervention website or wait-list control. Pre-intervention and after 8 months of follow-up, each practice distributed exit cards (brief patient surveys assessing provider performance, completed immediately after the dental visit) to 100 patients. Based on these exit cards, we assessed: whether patients were asked about tobacco use (ASK) and, among tobacco users, whether they were advised to quit tobacco (ADVISE). All intervention practices with follow-up exit card data were analyzed as randomized regardless of whether they participated in the Internet-delivered intervention.

**Results:**

Of the 190 practices randomized, 143 (75%) dental practices provided follow-up data. Intervention practices’ mean performance improved post-intervention by 4% on ASK (29% baseline, adjusted odds ratio = 1.29 [95% CI 1.17-1.42]), and by 11% on ADVISE (44% baseline, OR = 1.55 [95% CI 1.28-1.87]). Control practices improved by 3% on ASK (Adj. OR 1.18 [95% CI 1.07-1.29]) and did not significantly improve in ADVISE. A significant group-by-time interaction effect indicated that intervention practices improved more over the study period than control practices for ADVISE (*P* = 0.042) but not for ASK.

**Conclusion:**

This low-intensity, easily disseminated intervention was successful in improving provider performance on advice to quit.

**Trial Registration:**

clinicaltrials.gov NCT00627185; http://clinicaltrials.gov/ct2/show/NCT00627185 (Archived by WebCite at http://www.webcitation.org/5c5Kugvzj)

## Introduction

Despite widespread acceptance of the evidence that tobacco use is the primary preventable cause of death, rates of this risky behavior have not substantially declined in the past 10 years [1]. A recent state of the science conference on tobacco cessation noted that several interventions to enhance tobacco cessation are underutilized [2].

Brief provider-delivered interventions, applied during clinical visits, are effective in increasing cessation. A recent meta-analysis of brief provider-delivered cessation advice reported a pooled odds of patient cessation of 1.74 (95% CI 1.48, 2.05), comparing intervention to control [3]. Studies included in this synthesis of brief cessation advice were frequently based on the current “5A’s” approach. The 5A’s (Ask, Advise, Assess, Assist, and Arrange follow-up) are recommended in the *Treating Tobacco Use and Dependence* guideline [4].

The dental visit is a unique but underused opportunity for tobacco control, despite evidence that brief provider advice delivered in the dental setting is effective in increasing tobacco cessation [5]. Block et al surveyed healthcare providers in 1999 and found that 69% of physicians report consistently assessing tobacco use among patients, compared with 32% of dentists [6]. Only 13% of physicians reported never intervening with tobacco users, but nearly half (49%) of dentists never intervened. In a more recent survey, dentists again did not routinely incorporate the assessment of tobacco smoking into their practices, with half of dentists reporting providing cessation advice at least 41% of the time [7]. Only 20% of dentists in a recent community survey were aware of the tobacco guideline [8]. Increasing diffusion and uptake of guideline-adherent approaches to reducing tobacco use, especially in dentistry, is essential.

Prior studies of guideline implementation strategies, such as educational outreach and didactic continuing medical education, have resulted in median absolute improvements ranging from 6 to 8% for a variety of processes of care [9,10]. Implementation strategies including videos, self-study materials, educational outreach, and workshops have been documented to improve cessation advice in dentistry [11-14]. Improvements in care have resulted from interventions which were at times quite costly to deploy [9] and had considerable marginal costs for material, personnel, and travel per practice. To maximize the reach of guideline implementation, educational and behavioral interventions designed to be readily available, consistently used, and deployed with minimal cost per practice are needed.

Recently, the Internet has been used to deliver educational interventions to increase guideline compliance [15-18] at low costs [19,20]. We developed an interactive, Internet-delivered intervention designed to educate providers in dental practices and to provide motivation and resources for increasing tobacco control. OralCancerPrevention.org, the resulting practice improvement intervention, was evaluated using a randomized trial to measure changes in guideline-adherent tobacco control practices [21]. We hypothesized that access to the interactive, Internet-delivered intervention would increase rates of tobacco-use screening and cessation advice for tobacco users, comparing intervention and control.

## Methods

### Study Design Overview, Setting, and Sample of Participating Dental Practices

We conducted a randomized trial among dental practices from Alabama, Georgia, Florida, and North Carolina, identified using mailing lists from dental licensure and the Dental PBRN, a dental practice-based research network [22]. PBRNs are "groups of primary care clinicians and practices working together to answer community-based health care questions and translate research findings into practice. PBRNs engage clinicians in quality improvement activities and an evidence-based culture in primary care practice" (Agency for Healthcare Research and Quality). The community-based dental practices have a varying number of providers and are based in a variety of settings (rural and urban). Beginning in January 2005 through February 2006, dental practices were recruited using a letter addressed to the dentist which advertised the study. For blinding purposes the letter did not mention tobacco control but identified the study as an evaluation of an “Online Study Club for Oral Cancer Prevention”. Face-to-face study clubs are frequent in dentistry and usually refer to a group of dental providers who gather to discuss clinical practice and the dental literature [23-25]. Eligible practices included general dentistry or periodontal practices which reported having Internet access in their practice (requirement of the study) and indicated an interest in participating.

Accounting for clustering of patients within practices, we calculated a sample size of 130 practices (65 per arm) would be needed to detect a difference of 10%, comparing intervention and control. Anticipating an attrition rate of 30%, our targeted recruitment goal was 190 practices. Dental practices which initially agreed to participate were required to complete a run-in phase of baseline data collection, including patient and practice data, and then they were randomized. From our initial recruitment pool, we randomized the first 190 practices which returned the baseline data. Practices were randomized to the intervention described below, or a control group using a permuted block randomization sequence generated by our biostatistician. As practices returned baseline data, allocation to intervention or control was performed using the predetermined randomization sequence by an analyst blinded to the results of the baseline data. The protocol was approved by the University of Alabama at Birmingham Institutional Review Board.

After the run-in phase and randomization, the dentist and staff of intervention practices were sent a letter with information about the website and log-on instructions. We then tracked each practice to determine who from the practice logged on, when they logged on, and the amount of time they spent visiting the site. To encourage participation, emails were sent to the intervention participants alerting them about new website content and updates in the field of dental tobacco control. Once a practice logged on to the website, patient education materials about tobacco use were mailed to the practice for in-office use.

We used a delayed-intervention control group. Control practices continued to provide the usual care that they delivered to patients during the intervention period while still completing baseline and follow-up data collection. Control practices did not receive access to the intervention until all data collection was complete.

### Development of OralCancerPrevention.org—The Practice Improvement Intervention

We developed an Internet-delivered educational intervention designed to support oral cancer prevention in dentistry. The development team included a hygienist, dentist, and tobacco control and health informatics experts. Prior to the development of the website, we conducted 3 Nominal Group Technique (NGT) meetings, 2 with a total of 13 dentists, and 1 with 10 hygienists participating. The NGT is a structured approach to collecting and prioritizing input from stakeholders [26]. The question for NGT discussion (“What sorts of things could be done to ensure that as a routine part of every dental visit all patients are asked about their tobacco use and/or advised to quit using tobacco?”) was identified through numerous brainstorming sessions with the investigative team. The dentists and hygienists identified 76 potential strategies for promoting tobacco control, including 9 distinct educational issues. Based on the NGT findings, the investigative team along with programmers met weekly for 12 months to develop both the content and format of the Internet intervention, which resulted in an interactive, multi-component website with supporting emails. Usability testing was conducted to confirm ease of navigation. The site was designed to be accessed longitudinally over 8 months and be frequently updated with new content.

### OralCancerPrevention.org Content

The final Oral Cancer Prevention product was comprised of 3 educational cases, patient education and practice tools, a forum for chatting, opportunities to ask questions, and presentation of headlines (see Multimedia Appendix 1 for sitemap and screenshots). The dentist could spend from as little as a few minutes up to hours on the website. All course materials were updated as needed and the 3 cases were released at 2-month intervals.

The interactive educational cases were interspersed at key decision points with questions, and we provided targeted feedback based on user responses. In addition, references and literature were available at critical points to support the course material. Dentists and hygienists could access downloadable, patient education materials and practice tools, including brochures and posters. A discussion forum allowed the dentists and dental staff to post questions and receive feedback/responses from other dental staff and practitioners. The “ask a question” feature allowed any participant to submit a question related to oral cancer prevention and receive a direct response from the investigative team. In addition, we emailed all participants bi-weekly with “headlines” presenting new research findings to the group and/or with “questions of the week” asking challenging questions related to tobacco control.

Participants received one continuing education unit for each of the cases completed. As cues to log on, we provided the practices with calendars, pens, and squeeze balls that had the website address and the project name. The intervention was available over an 8-month period for each practice.

### Baseline Practice Variables and Longitudinal Tracking of Participation

Data were collected from the practices at baseline before randomization. The baseline practice survey included an assessment of the number of dentists, hygienists, and dental assistants in the practice; the number of years employed at that practice; and current oral cancer prevention-related activities.

Once randomized, user authentication was required for all providers as they logged onto the intervention. This allowed use of server tracking logs linked to site visits to measure participation. The administrative portal of the study website tracked type of page visited, volume of pages, number of visits by practice and individual, date of access, time of access, name, and practice identification of each participant who logged onto the site [27]. We used the total number of pages of website content accessed as a marker for overall participation.

### Measuring Provider Performance of Tobacco Control Activities (Main Outcome)

Our main outcome measures were based on the patient reports of guideline-compliant provider performance of tobacco control for the first 2 components of the 5A’s (ASK and ADVISE) [4]. The 2 main outcomes were the proportion of patients asked if they were tobacco users (ASK), and among the tobacco users, the proportion who were advised to quit (ADVISE). Accordingly, we collected patient reports of provider behaviors using patient exit cards.

After completing the practice survey, practices were provided a set of 100 patient exit cards. The patient exit cards, brief post-card sized surveys, were completed by adult patients at the end of their appointments prior to leaving the office. The exit cards were developed using principles of ecological momentary assessment (EMA) [28-30]. First, EMA is completed as close in time to the exposure as possible to avoid faulty recall. Second, EMA is designed to be brief and unobtrusive to maximize participation rates and diffusion.

The exit cards were designed to be completed in 1 to 2 minutes while the patient was awaiting follow-up instructions and completing payment. Each practice was provided with instructions to hand out these exit cards to 100 consecutive adult patients after their visit. Each patient was provided a pen to complete the survey, and they were allowed to take the pen as a gift. Patients completing cards then deposited them in a sealed collection box. When all 100 cards were distributed, the dental practice returned the collection box to our coordinating center.

We used the patient exit card to assess patient tobacco use, age, and gender. Patients indicated whether they had been asked about tobacco use and, if a tobacco user, whether they had been advised to quit. To blind the patient and practice to the outcome of interest, the exit card also included questions related to alcohol use and counseling, as well as dietary intake and counseling, received at the practice. Patients indicated on the card if they were willing to be contacted for a follow-up call and, if so, provided their name and telephone number. A sample of 150 patients from 6 practices was called to evaluate the reliability of the patient exit card data [31]. Agreement between card and telephone interview responses on whether the patient was a tobacco user was high (99%), with only 2 disagreements. Agreement rates for patient age and gender comparing immediate and delayed were also high (97% and 100%, respectively).

### Statistical Analysis

In this trial, the unit of randomization was the dental practice, and both dentists and their staff were the targets of the intervention. Our analysis used an intent-to-treat design including all practices with follow-up data available. Intervention practices were analyzed as randomized regardless of whether they actually used the intervention. As noted, dependent variables for this study are patient-reported provider performance measures (ASK and ADVISE) collected as binary variables at the patient level. As patients were clustered within practices and the unit of randomization was at the practice level, we used a modeling approach appropriate to hierarchical data. Common approaches to clustered data include generalized estimating equations and generalized linear mixed models [32,33]. As the number of smokers per practice varied, we chose to use a generalized linear mixed model approach with adaptive quadrature with a logit link for binary outcomes because this approach is more robust to variations in intra-class correlation coefficient and cluster size [34,35]. This analysis was implemented using the Generalized Linear Latent and Mixed Models (GLLAMM) procedure in the STATA software package and verified using the SAS software package.

To assess the impact of the intervention, we first calculated the unadjusted proportion of patients who were asked and smokers who were advised pre- and post-intervention. Then, separately for intervention and control, we assessed the difference pre- versus post-intervention. For each indicator (ASK and ADVISE), we developed 2 models (1 for intervention, 1 for control). Finally, significance of differences (pre- versus post-intervention) in the odds of patient reports of ASK or ADVISE in intervention versus control practices were determined. For each indicator (ASK, ADVISE), 1 overall model, including both control and intervention patients, was developed. We included a term for Group (intervention versus control) and Time (pre- versus post-intervention), as well as a group-by-time interaction term. Significance of differences in improvement over time, by group, was determined by the statistical significance of the group-by-time interaction term in these overall models.

As a secondary analysis, we conducted a “per protocol” analysis excluding intervention-arm practices that did not participate in the intervention to further estimate what the optimal effects might be for this Internet-delivered intervention.

Because participation in Internet-delivered interventions such as this is inherently variable, we further assessed a dose-response by level of participation among intervention practices.

## Results

### Participating Practices

**Figure 1:**
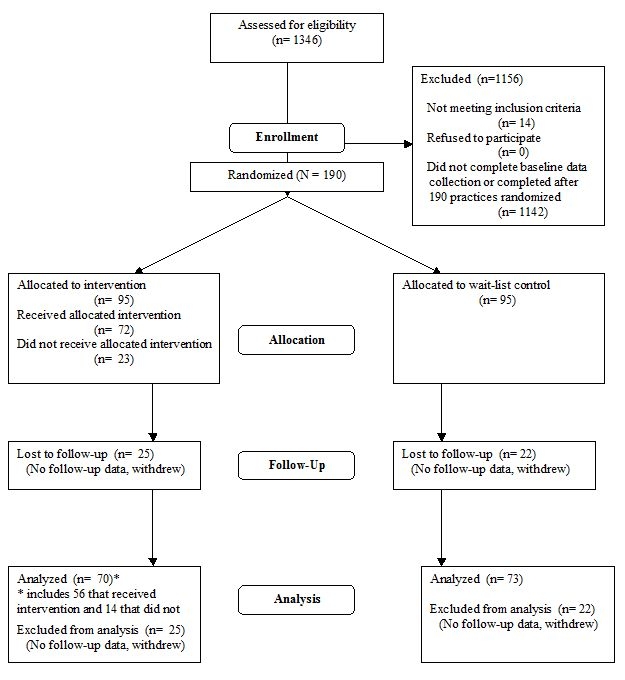
CONSORT Flowdiagram: Recruitment and retention of dental practices

From a group of 1346 practices initially expressing interest, we randomized the first 190 practices that completed data collection. Of the 190 dental practices randomized, 75% (143) completed follow-up data collection (see Figure 1).

Most of the 143 practices were general dentistry practices (92%) and solo practices (79%). Practices were located in Alabama (25%), Florida (34%), Georgia (27%), and North Carolina (14%). Overall, these 143 practices included 185 dentists (89 intervention and 96 control) and 274 hygienist participants (137 intervention and 137 control). Practices varied in the number of support staff, with most having 3 or more dental hygienists and dental assistants (Table 1). Overall, these were fairly established practices. In 83%, the primary dentist had practiced there for over 5 years. Control practices had a mean of 4.5 (SD 3.7) Internet-accessible computers, and intervention practices had 4.3 (SD 3.9) (*P* = 0.41). Comparing intervention and control practices, we found no differences in these characteristics at the *P* < 0.05 level. Providers also reported the overall characteristics of their patients, including the proportion of patients who were minorities (mean = 32%, SD 24), the proportion who had dental insurance (mean = 31%, SD 19), and the proportion who were on public assistance (mean = 11%, SD 21). Practices characteristics’ and baseline provider performance were similar among those that completed follow-up and those that did not (summary data available in Multimedia Appendix 2).

**Table 1:** Characteristics of 143 dental practices randomized to intervention or control with completed follow-up^a^

	Control		Intervention	
	n/N^b^	%	n/N^b^	%
**Practice Type**				
General Practice Periodontal	69/73 4/73	94.5 5.5	63/70 7/70	90.0 10.0
**Solo/Group Practice**				
Solo Dental Practice Group Dental Practice	57/72 15/72	79.2 20.8	53/68 15/68	77.9 22.1
**Number of Hygienists and Assistants**				
0 staff 1-2 staff 3-4 staff >4 staff	1/73 17/73 39/73 16/73	1.4 23.3 53.4 21.9	3/70 20/70 26/70 21/70	4.3 28.6 37.1 30.0
**Number of Years at this practice(Dentist)**				
<5 years 5-10 years >10 years	11/66 13/66 42/66	16.7 19.7 63.6	12/66 12/66 42/66	18.2 18.2 63.6
**Urban or Non-urban**				
Urban over 1 million Other metro Non-metro	26/73 35/73 12/73	35.6 48.0 16.4	22/70 36/70 12/70	31.4 51.4 17.1
**Practice busyness**				
Too busy to treat all Overburdened Not overburdened Not busy enough	6/72 7/72 50/72 9/72	8.3 9.7 69.4 12.5	9/70 6/70 45/70 10/70	12.9 8.6 64.3 14.3
**State**				
AL FL GA NC	25/73 20/73 18/73 10/73	34.3 27.4 24.7 13.7	11/70 28/70 21/70 10/70	15.7 40.0 30.0 14.3
**Number of Patients Visits Per Week**				
<=40 patients/week 40-100 patients/week >100 patients/week	8/73 47/73 18/73	11.0 64.4 24.7	4/70 47/70 19/70	5.7 67.1 27.1

^a^ No significant differences in practice characteristics between intervention and control were found (all *P* >0.05)

^b^ Denominator varies slightly due to small number of missing data

### Participation in the Internet-Delivered Intervention

**Figure 2:**
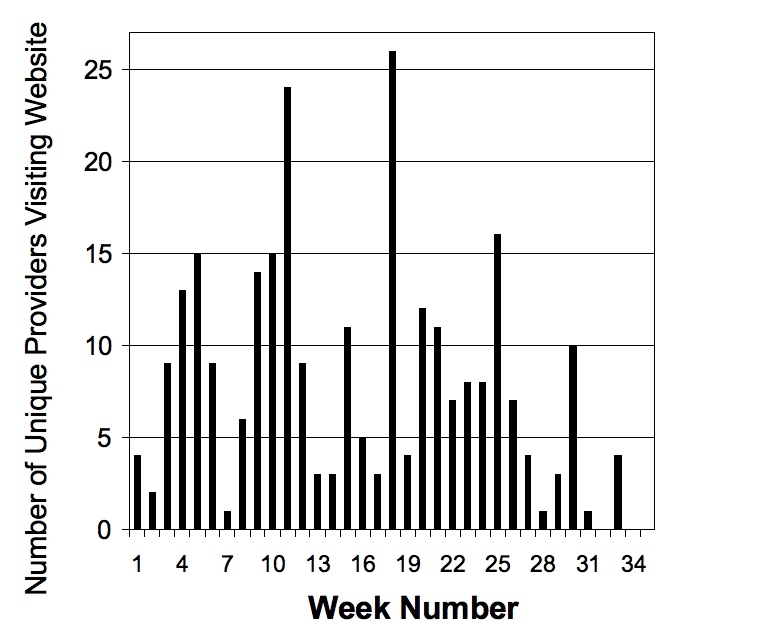
Number of unique providers visiting the website per week over 8 months

Of the 70 intervention practices that participated in follow-up, 56 (80%) had at least 1 provider who actually participated in the intervention. In the 56 participating practices, 53 of the 56 dentists (95%) and 38 of the 56 hygienists (68%) logged on to the intervention website. The mean number of tracked pages per practice was 50 (SD 40), and these ranged from 1 to 157. The mean number of visits to the intervention per practice that logged on was 5.8 (SD 4.6), and the mean number of unique participants was 1.9 (SD 1.2), ranging as high as 6 participants (dentists and hygienists) in a single practice. Figure 2 displays the number of unique providers visiting the website per week of intervention time. The spikes in activity centered at weeks 12 and 18 correspond to the initial release of additional interactive cases. Other smaller spikes represent response to headlines and questions-of-the-week updates. For the 3 cases, 75% (42/56) of practices had at least 1 provider complete Case 1, 55% (31/56) had at least 1 provider complete Case 2, and 21% (12/56) completed Case 3.

### Patient-Reported Provider Performance on “ASK” and “ADVISE” Before Intervention

Of the 14,300 pre-intervention exit cards distributed to these 143 practices, 11,898 (84%) were returned completed. Intervention patients completing the cards had a mean age of 48 (SD 14), and control patients had a mean age of 49 (SD 16). Both groups were 61% female. Of the 11,898, 21.3% were tobacco users.

At the patient level, of the 11,898 patients, 3421 (28.8%) reported being asked about tobacco use at their current visit. Among the 2386 tobacco users, 43% reported being advised to quit. At the practice level (Table 2), pre-intervention performance, as measured by mean proportion of patients reporting ASK and ADVISE, was similar between intervention and control practices and was not significantly different after accounting for clustering using GLLAMM.

### Patient Reported Provider Performance After Intervention (Intent to Treat)

At the patient level, for these 143 post-intervention practices, the exit-card response rate was 81.6% (11,678/14,300). Patient characteristics for this cohort were similar to the pre-intervention group, with a mean age of 47.5 years (SD 16), 59.3% being female, and 22.6% being smokers.

In adjusted analysis, accounting for clustering of patients within practices, both intervention and control improved slightly for ASK, but their rates of change over time, as measured by the group-by-time interaction term, did not differ significantly (Table 2). Intervention practices improved on ADVISE significantly more than control practices (*P*-value for the interaction term = 0.01).

**Table 2:** Odds of receiving screening and advice to quit smoking among patients in 143 intervention and control practices, comparing pre- and post-intervention

	Control (N = 73 practices)						Intervention (N = 70 practices)						Intervention Versus Control
Provider Performance	Pre- Intervention		Post-Intervention		Adj. Odds Ratio^a^ (95% CI) [ICC] ^b^		Pre-Intervention		Post-Intervention		Adj. Odds Ratio^a^ (95% CI) [ICC] ^b^		Group X Time *P* value^c^
	n/Total N	(%)	n/Total N	(%)			n/Total N	(%)	n/Total N	(%)			
ASK (Tobacco Use Screening)	1,693/ 6,080	27.8	1,794/ 5,759	31.2	1.18	(1.07-1.29) [0.21]	1,728/ 5,818	29.7	1,957/ 5,744	34.0	1.29	(1.17-1.42) [0.30]	0.19
ADVISE (Tobacco Use Counseling)	488/ 1,169	41.8	545/1210	45.0	1.13	(0.89-1.43) [0.09]	529/ 1,190	44.5	748/ 1,361	55.0	1.55	(1.28-1.87) [0.22]	0.01

^a^ Odds ratios for post-intervention versus pre-intervention with clustering of patients within practices modeled with a generalized linear mixed effects model with a logit link and adaptive quadrature implemented in STATA using GLLAMM and confirmed in SAS.

^b^ ICC = Intraclass Correlation Coefficient for practice-level effect.

^c^ *P* value from group-time interaction term included in a generalized linear mixed effects model with a logit link and adaptive quadrature implemented in STATA including intervention at control data from pre- and post-intervention. Results confirmed in SAS.

### Per-Protocol and Dose-Response Analyses

In our per-protocol analysis we kept only the intervention practices with follow-up data that actually logged on to the website at least once (N = 56) and compared them to the control practices. In this model, the effect of the intervention was strengthened with the cluster-adjusted odds ratios of receiving advice to quit post- versus pre-intervention being 1.74 (95% CI 1.42-2.12) for the intervention group (*P* for group by time interaction term = 0.004). Again, ASK was not significantly different when comparing intervention and control.

Within the intervention group, we found that greater participation in the intervention resulted in greater improvement, with increases in ADVISE of 4% among those who did not log on, 9% in those practices who viewed less than the median number of pages viewed, and 14% in those with the highest level of participation (above median). The cluster-adjusted odds ratios of patients receiving advice to quit post-intervention versus pre-intervention were 1.31 (0.88-1.34) for those intervention practices that did not log on, 1.59 (1.21-2.09) for those with less than the median number of pages viewed, and 1.92 (1.43 – 2.56) in those with the highest level of participation. Higher levels of participation were not associated with greater improvement in ASK.

## Discussion

The intervention had a strong effect, a 10% increase, on practice behavior related to delivery of advice to quit tobacco among tobacco users. Our study is the first to demonstrate that a multimodal, Internet-delivered intervention designed to promote and support tobacco control in dental practices can be effective. As with most Internet-delivered interventions, the website required a considerable start-up effort in terms of content development (intellectual content), web programming, and usability testing to ensure consistent navigation. However, the marginal server demands to disseminate the intervention to each additional practice were low.

For some online interventions directed at changing provider behavior, the evaluations have ended at changes in knowledge and attitudes [17,36-40]. Our goal was to directly assess changes in provider behavior as measured by patients. When provider performance outcomes have been assessed, results of Internet-delivered interventions for providers have been mixed [15,16,41,42]. In some of these interventions, baseline rates of provider behavior have been higher than anticipated, reducing the ability to affect change [16]. Our intervention clearly benefited from the fact that there was clear room for improvement in targeted behaviors.

Baseline rates of ASK in our sample were less than 30%, and ADVISE was 42% in control and 44% in intervention. In prior studies, rates of ADVISE in dental practices varied from 30% to 50%, depending on the setting, sample, and respondent (patient or provider) [6,7,31,43-45]. In a randomized trial, Andrews et al reported that patient-reported control group rates of dental provider advice to quit were 42.4%, which is similar to our findings [43].

We were successful in engaging 80% of the intervention practices in the website activities, and among those practices that did participate, a high proportion of dentists and hygienists logged on. Low rates of participation have been sighted as a reason for limited success in some Internet-delivered interventions targeting providers [41]. Of note, our intent-to-treat analysis demonstrated an impact of the intervention even though 20% of the intervention practices did not use the website cases and supportive tools. Among those practices that did participate, we were moderately successful in sustaining activity over 8 months. Previous research in online professional development suggests that a “spaced education” approach, where content is distributed, repeated, and reinforced over time, has a stronger impact on knowledge and subsequent behavior than a one-time education [46]. We used automated reminders and frequent content updates that served as hooks to encourage repeated participation over the 8 months.

Our study has several limitations. As noted, we recruited our 190 dental practices from a large pool of practices. We required a run-in phase and enrolled the first 190 practices who completed the baseline data collection. Although not uncommon in randomized trials, the low enrollment to recruitment ratio suggests that our practices may be somewhat different than the average dental practice. Specifically, these practices may be more computer-oriented and more Internet-savvy than the average practice. Attrition was also a limitation. In terms of the outcome of interest, a direct measurement of provider behavior, such as audiotapes of visits or direct observation, was not accomplished nor was it feasible in a study of this size. We demonstrated that distribution and collection of exit cards from patients was feasible, and that the office staff was willing to support the study with a small incentive for data collection. As discussed above, we validated the results of the exit cards with patient phone calls in a subset.

In our study, rates of advice to quit smoking increased 10% in intervention practices with only marginal increases in patient reports of being asked about tobacco use by a provider. Tobacco control guidelines emphasize the need for systematic screening as a first step in tobacco control that leads to increasing advice [4]. Some studies in medical practice suggest that screening increases advice [47,48]. In preliminary nominal group technique meetings, dentists reported that they could often “tell” that patients were tobacco users without asking. It may be that through the oral exam and having a working space that is close to the patient's face, dental providers are able to more accurately diagnose tobacco use in the absence of screening than medical providers [49]. The oral exam itself may provide a strong cue to delivering quit tobacco advice. If active screening had been implemented by the dental providers, we may have seen an even greater increase in cessation advice.

We chose to assess provider performance based on patient reports collected immediately after the visit. Assessments of provider delivery of tobacco control services are increasing [50-58]. Patient reports of provider behavior have been used for outcome assessments such as ours [51-55,57,58]. Compared to the gold standard of audio-tapes of doctor-patient encounters, immediate surveys of patients are more accurate than provider reports or chart abstraction [51,52,57]. The Health Plan Employer Data and Information Set (HEDIS), a set of standardized performance measures collected by the National Committee for Quality Assurance, adopted patient-report of provider tobacco cessation advice as a national standard [59]. 

In conclusion, the intervention was successful, but success was somewhat limited by initial participation in the intervention and waning activity over time. Future intervention activities should include additional marketing and persuasive techniques to encourage and sustain participation. We interpret the results of this study to suggest that dental practices are settings where low-intensity interventions to support tobacco control can be effective. The Internet-delivered intervention in this study was more successful than some prior interventions in medical practice, also supporting the potential of the Internet for outreach in dentistry.

## Multimedia Appendix 1

Sitemap and screenshots of www.oralcancerprevention.org [21]

## Multimedia Appendix 2

Supplemental data tables
