# Supplementary material for: Internet Delivered Support for Tobacco Control in Dental Practice: Randomized Controlled Trial
Source: J Med Internet Res. 2008 Nov 4;10(5):e38. doi: 10.2196/jmir.1095 (PMC2630831; doi:10.2196/jmir.1095)
Supplement: Supplementary file 1 [file jmir_v10i5e38_app1.pdf]

Internet Delivered Support for Tobacco Control in Dental Practice: Randomized Controlled Trial
J Med Internet Res 2008;10(5):e38

Multimedia Appendix 1: Sitemap and screenshots of www.oralcancerprevention.org

Sitemap of www.oralcancerprevention.org

Oral Cancer Prevention Project Website Homepage

SECTION 1:
Educational Cases

Case 1
Goal: How to approach tobacco screening during a routine dental exam
Health History Form
Ask Advise Refer
Personal Relevance
5 R's
Print your CME

Case 2
Goal: How to assess a patient's readiness to quit tobacco and how to assist a patient in quitting
Screening at each visit
Assess Readiness to Quit
Identify appropriate messages for precontemplator
Assist the preparation patient to quit
Print your CME

Case 3
Goal: How to encourage and assist a patient who is ready to quit tobacco
Personal Relevance
Assist the preparation patient to quit
Print you CME

SECTION 2:
Patient Education and Practice Tools

1. Patient Education Materials
Quit Smoking Rationale Card
Social Contract
What the patient can expect
Distraction and Substitution
You Can Quit Smoking, pocket card
You Can Quit Smoking, 5 day Countdown
You can Quit Smoking, consumer guide
Support and Advice From Your Clinician
Support and Advice From Your Prenatal Car Provider

2. Practice Tools
Dental Practice Tobacco Use Cessation Protocols
Smoking as Vital Sign
5A's Intervention
Tobacco Cessation F/U Record and Index Card

3. Help Your Patients Quit – The Quitting Toolbox

4. Patient Quit Line (1-800-Quit-Now)

5. Information about TREATMENTS
Nicotine Replacement
Commit Lozenge
Buproprion
Nortryptyline
Table Comparing Treatments

6. Library for your practice
Smoking and Risk to Oral Health
Smoking and Risk to General Health
How to Counsel- the 5A's and 5R's
Tobacco Control in Dental Practice
Do Dental Patient Expect Counseling?
Headlines

7. The Treating Tobacco Use & Dependence Guideline

8. Oral Cancer Screening Exam Poster Checklist

SECTION 3:
Other Supplementary Content

Forum: chat with others
Discuss the content of the study club and other dentistry-related issues with providers participating in the study club.

Ask-A-Question
Ask questions to project coordinators regarding oral cancer prevention.

Headlines
Read current research and news in oral cancer prevention in dentistry.

Provider's Stories
Read how other providers have made oral cancer prevention work in their practice.

Screenshot 1: www.oralcancerprevention.org home page

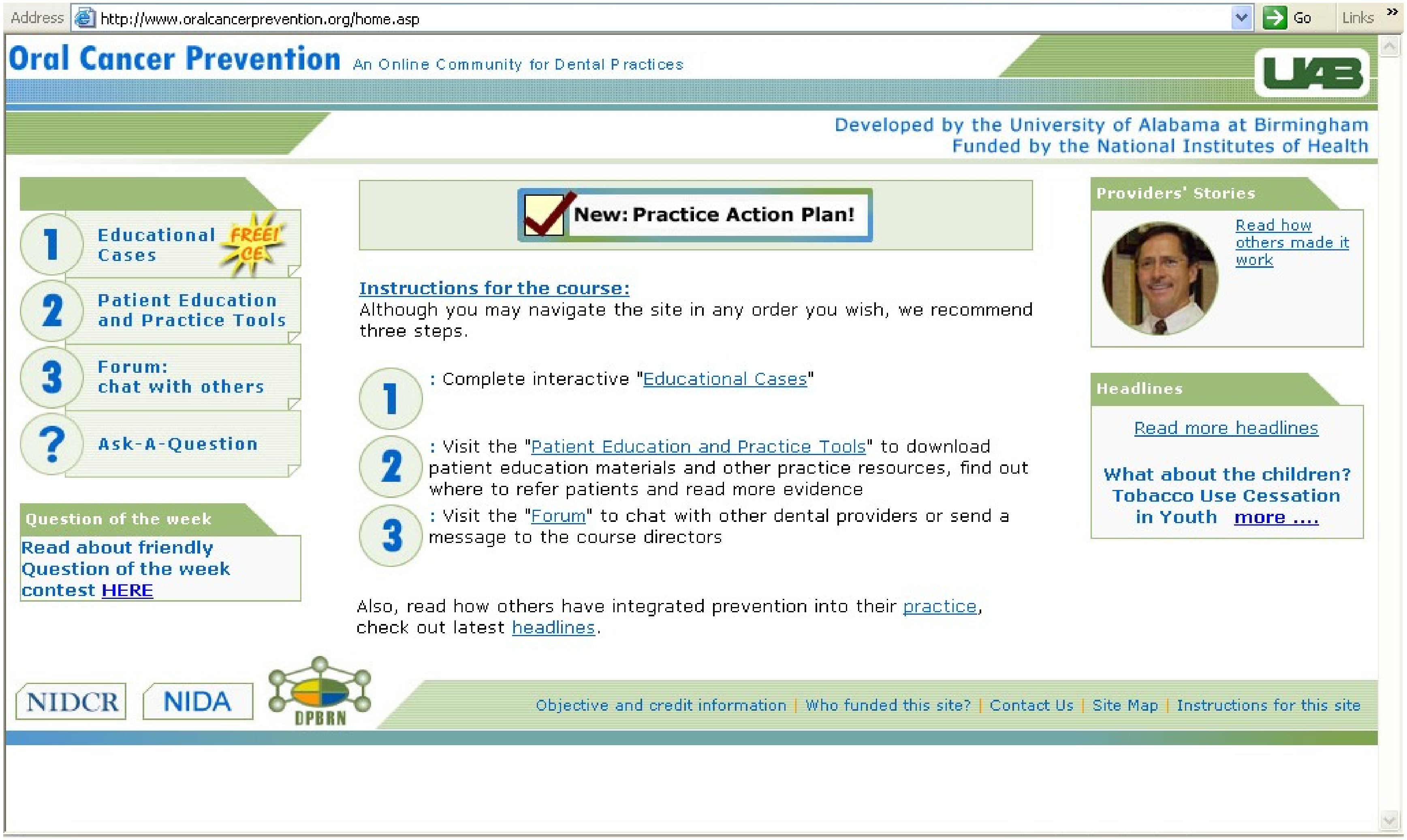

Screenshot 2: Question 1 of CASE 1

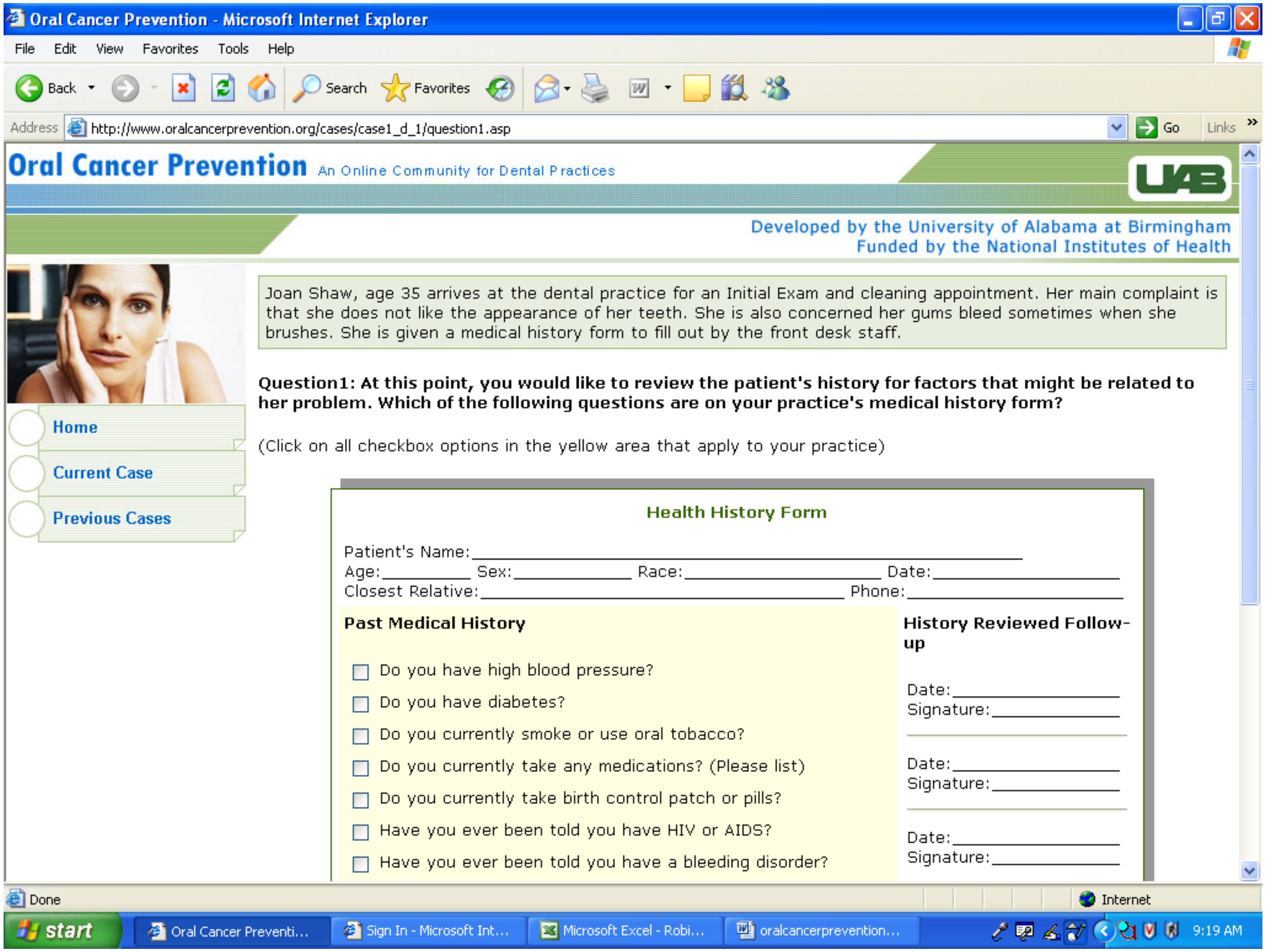

Screenshot 3: Example tailored feedback to Question 1 Answer

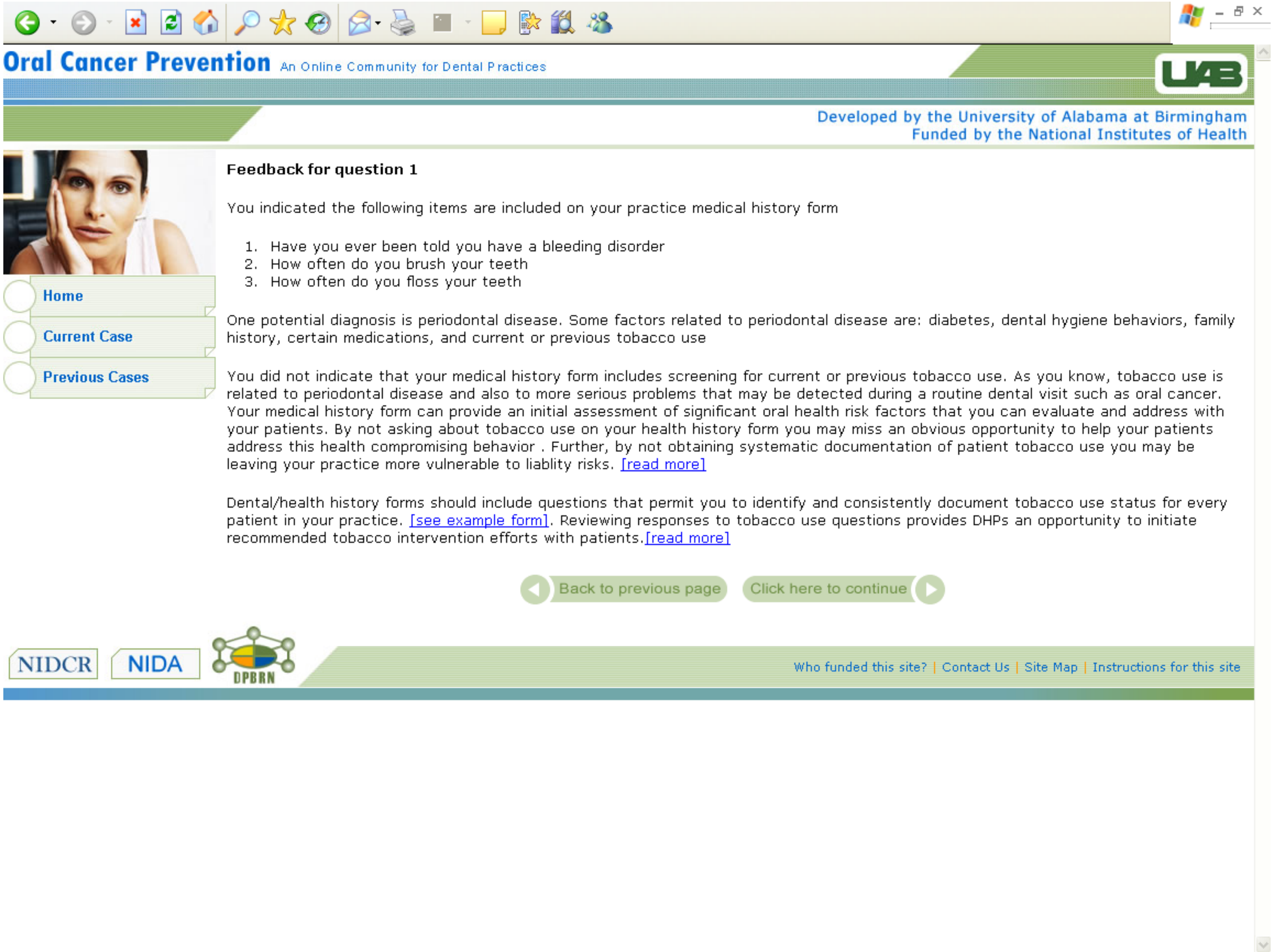

Screenshot 4: Example of Toolbox Page

Address http://www.oralcancerprevention.org/toolbox/patientEd/default.asp Go Links

Oral Cancer Prevention

An Online Community for Dental Practices

Developed by the University of Alabama at Birmingham
Funded by the National Institutes of Health

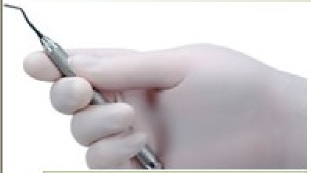

- Home
- Back to Patient Education and Practice Tools
- Patient Education Materials
- Practice Tools
- Help Your Patients Quit
- Patient Quit Line
- Preview of Patient Website
- Information about TREATMENTS
- Library for your practice
- The Guideline

Patient Education Materials

Click on the right links below. Download and save the patient education materials so you can print them out. The materials are available in both Word document and Adobe PDF formats, and will open in a separate window. (close the window to return here).

You can also contact Andrea Mathews at (205) 934-5816 and we can mail you these supplies.

1. Quit Smoking Rationale Card

Word Document format
(file size = 29 Kb, 8 cards per page)

PDF format
(file size = 10 Kb, 8 cards per page)

Request color copies

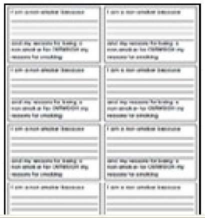

Screenshot 5: Further Example of Toolbox Pages (QuitLine link)

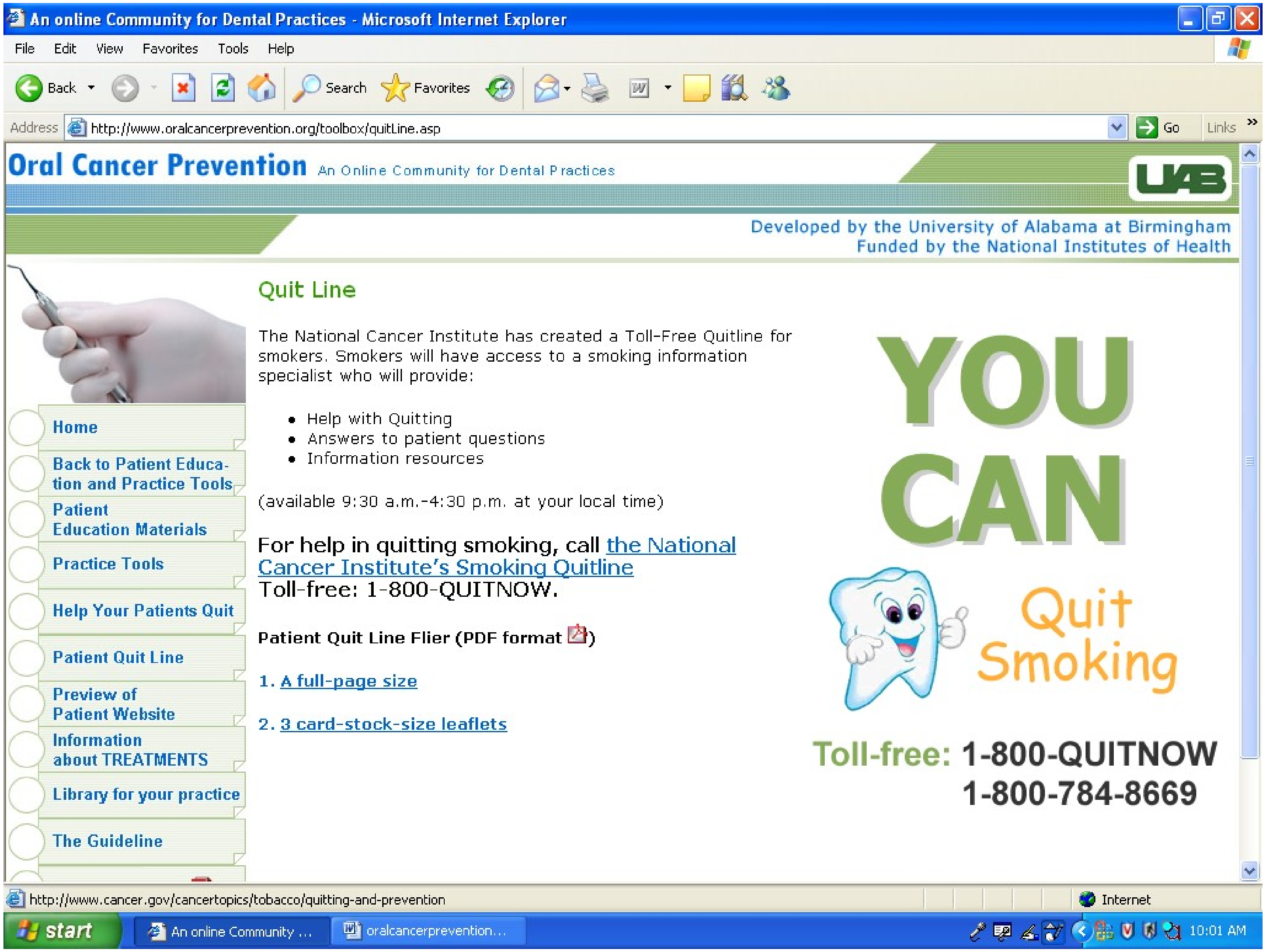

Screenshot 7: Further Example of Toolbox Pages

Address http://www.oralcancerprevention.org/toolbox/helptoquit/helptoquit.asp Go Links

Oral Cancer Prevention An Online Community for Dental Practices

UAB

Developed by the University of Alabama at Birmingham
Funded by the National Institutes of Health

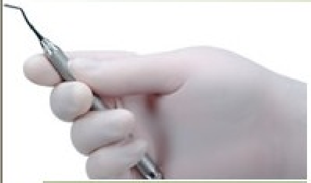

- Home
- Back to Patient Education and Practice Tools
- Patient Education Materials
- Practice Tools
- Help Your Patients Quit
- Patient Quit Line
- Preview of Patient Website
- Information about TREATMENTS
- Library for your practice
- The Guideline

The Quitting Toolbox

As your patient is preparing to quit, here are some strategies for coping with cravings and difficult situations that might lead them to smoking.

The urge to smoke may last 2-3 minutes and your patient cannot rely on willpower alone to get through the urge. Urges happen many times during the day so they will need to develop a plan to help get through them. This is where you can help.

Your patient needs to identify the factors that trigger their urges. Some common triggers are:

- Frustration
- Depression
- Stress/Anxiety
- Spouse, close friend, or people around you are smoking
- Craving a cigarette after coffee or eating

Once your patient identifies the factors, they need to come up with strategies to help them cope with their urges.

Here are some strategies that your patient could use as coping tools until they get more independent of the urges.

- **Self Talk** – What your patient thinks or says to themselves can play a big role in how hard or how easy it is for them to quit smoking. Tell your patient that by thinking and speaking positively they will have better control over their urges. When your patient recognizes that they are having negative thoughts, tell them to interrupt it and replace it with a positive statement. **EX.** I can handle just one cigarette.

  STOP AND THINK. "This urge will pass soon. If I smoke now, I will regret it later."

- **Relaxation** – A coping tool used to reverse the stress of quitting. Relaxation is used to reverse the body's

Done Internet
