# Supplementary material for: Internet Delivered Support for Tobacco Control in Dental Practice: Randomized Controlled Trial
Source: J Med Internet Res. 2008 Nov 4;10(5):e38. doi: 10.2196/jmir.1095 (PMC2630831; doi:10.2196/jmir.1095)
Supplement: Supplementary file 2 [file jmir_v10i5e38_app2.pdf]

Internet Delivered Support for Tobacco Control in Dental Practice: Randomized Controlled Trial
J Med Internet Res 2008;10(5):e38

Multimedia Appendix 2: Supplemental data tables

Table 1: Practice Characteristics of 143 practices with and 47 practices without follow-data

	With Follow Up (N=143)		Without Follow Up (N=47)	
	n/N	%	n/N	%
**Practice Type**				
General Practice	132/143	92.3	44/47	93.6
Periodontal	11/143	7.7	3/47	6.4
**Solo/Group Practice**				
Solo Dental Practice	110/143	78.6	36/47	76.6
Group Dental Practice	30/143	21.4	11/47	23.4
**Number of Hygienists and Assistants**				
0 staff	4/143	2.8	2/47	4.3
1-2 staff	37/143	25.9	13/47	27.7
3-4 staff	65/143	45.5	20/47	42.6
>4 staff	37/143	25.9	12/47	25.5
**Number of Years at this practice(Dentist)**				
<5 years	23/143	17.4	7/47	15.9
5-10 years	25/143	18.9	6/47	13.6
>10 years	84/143	63.6	31/47	70.5
**Urban or Non-urban**				
Urban over 1 million	48/143	33.6	17/47	36.2
Other metro	71/143	49.7	22/47	46.8
Non-metro	24/143	16.8	8/47	17.0
**Practice busyness**				
Too busy to treat all	15/143	10.6	5/47	10.9
Overburdened	13/143	9.2	7/47	15.2
Not overburdened	95/143	66.9	24/47	52.2
Not busy enough	19/143	13.4	10/47	21.7
**State**				
AL	36/143	25.2	10/47	21.3
FL	48/143	33.6	19/47	40.4
GA	39/143	27.3	12/47	25.5
NC	20/143	14.0	6/47	12.8
**Number of Patients Visits Per Week**				
<=40 patients/week	12/143	8.4	7/47	14.9
40-100 patients/week	94/143	65.7	28/47	59.6
>100 patients/week	37/143	25.9	12/47	25.5

Note: No comparisons are significant at p=0.05 level
Data also available at http://www.dpbrn.org/users/publications/Supplement.aspx

Table 2: Pre-intervention patient-reported provider performance comparing 143 practices with and 47 without Wave 2 Data

	**With Follow Up (N=143 practices)**		**Without Follow Up (N=47 practices)**	
	**n/N**	**%**	**n/N**	**%**
ASK_SMOKE	3421/11898	28.8	1158/3741	31.0
ADVISE_SMOKE	1377/3287	41.9	464/1119	41.5

Data also available at http://www.dpbrn.org/users/publications/Supplement.aspx
